# Investigation of the Cutting Fluid Incidence Angle Direction in Turning Grade 5 ELI Titanium Alloy under High-Pressure Cooling Conditions

**DOI:** 10.3390/ma16155371

**Published:** 2023-07-31

**Authors:** Grzegorz Struzikiewicz

**Affiliations:** Faculty of Mechanical Engineering and Robotics, AGH University of Science and Technology, 30-059 Cracow, Poland; gstruzik@agh.edu.pl

**Keywords:** titanium alloy Grade 5 ELI, high-pressure cooling, turning, cutting forces, chip breaking index

## Abstract

The use of high-pressure cooling (HPC) in machining can increase the efficiency and improve process stability through more effective breaking and chip evacuation. Turning tests of the Grade 5 ELI titanium alloy were carried out using cemented carbide tools and taking into account the direction of feeding of the cutting liquid. Measurements of the components of the total cutting force were carried out for feeds in the range *f* = <0.08; 0.13> mm/rev and two angular settings (i.e., angle α = <30°; 90°> and β = <0°; 60°>) of the nozzle. The chip breakage coefficient was determined. It was shown that the cutting force values depended on the feed value, and the angle of feeding of the cutting fluid did not significantly affect the values of the cutting forces. Despite the different forms of chips obtained, the applied method of searching for the best conditions was unsuccessful and no significant effect on the values of the chip breaking coefficient *Cch* was observed. To determine the best nozzle setting, it is useful to determine the working area of the chip breaker. Due to the shape of the chip, the optimal angular setting for the nozzle that supplied the cutting fluid was α = 60° and β = 30°. In addition, it was observed that the angle of incidence of the cutting fluid jet could affect the chip formation process and support the chip cracking process.

## 1. Introduction

The machining of titanium alloys is a topical issue, especially in the context of their production and operation. The optimisation of existing machining processes and the development of new techniques to ensure the expected machining efficiency with the desired quality and low costs are the main directions of research for difficult-to-cut materials, including titanium alloys [1,2]. Difficult-to-cut materials are materials with special properties, such as mechanical properties, thermal conductivity, or chemical reactivity. This group of materials includes the so-called nickel- and cobalt-based superalloys, titanium alloys, and heat-resistant steels [3,4,5]. Titanium alloys are used most frequently by the chemical and marine industries due to their excellent corrosion resistance [1,2]. Because it is an inert material in contact with biological tissue, it is used in the medical and dental industry for surgical and prosthetic implants [6]. Ti6Al4V-ELI (Extra Low Interstitials), also called Grade 5 ELI titanium alloy, contains less oxygen, nitrogen, carbon, and iron than a typical Ti6Al4V alloy. The increase in the percentage of these elements improves the ductility and fracture resistance of the material. These particular properties mean that this alloy is used in dentistry and medicine, e.g., for orthopaedic implants [6,7,8]. In turn, the high strength-to-weight ratio of titanium alloys allows their use in the auto- and aero-industries [2]. Despite the known causes and difficulties repeatedly described in the literature, research is still being carried out to broaden knowledge in the machining of titanium alloys. The area of research focused on the machining of titanium alloys is mainly concerned with the influence of the cutting parameters on the quality of the machined surface and the determination of the values of the forces and temperature in the cutting zone [3]. Another area of research concerns the issues of the process of chip formation and cracking during machining, as well as their computer simulation [9]. An overview of the listed issues is briefly presented in the text below.

Due to the high temperature values in the cutting zone and the stress concentration at the edge of the cutting edge, approximately 80% of the heat is supplied and transferred by the cutting tool [3,10]. This is because of the low thermal conductivity value, which causes accelerated tool wear during cutting. To improve the machinability of titanium alloys, various cooling machining techniques have been developed: minimum quantity lubrication (MQL) [11,12], high-pressure cooling (HPC) [13,14], or cryo-machining [15,16].

A frequently discussed issue by researchers is machining under the elevated pressure of the cutting fluid fed into the cutting zone. Currently used systems enable the feeding of the cutting fluid with a pressure from 50 to even 1000 bar. Compared to conventional cooling, HPC-based machining can use a higher cutting speed, which increases the production efficiency. Moreover, by reducing the temperature in the cutting zone, the tool life can be increased by 5 to 15 times. However, the basic application of this method is to support the chip breaking process. Not without significance is also the increase in chip removal efficiency outside the machining zone [17,18].

In the field of turning titanium alloys, Palanisamy et al. [18] described a series of experiments at different cutting parameters and pressure levels for carbide insert machining. Studies have shown that the use of a high-pressure cutting fluid improves the process of breaking and removing chips from the cutting zone. The reason is the mechanical impact of the liquid stream on the chips formed. They also found that HPC machining increases the tool life by almost three times compared to conventional cooling. In turn, Ezugwu et al. [19] comparatively analysed the machinability of a titanium alloy under conventional and high-pressure cooling conditions in the machining zone. They analysed the influence of different cutting parameters using tools made of cemented carbides coated with different coatings. The use of a high pressure reduces the cutting force components, ensures the desired brittleness of the chip, and results in less wear to the cutting tool. Stolf et al. [20] also analysed the wear mechanism in terms of tool–chip contact conditions when machining Ti6Al4V with HPC supply. They concluded that the coolant pressure and the maximum flank wear were inversely proportional to each other, indicating an influence of the heat directed towards the flank face of the cutting tool on the abrasion process. The experiments also showed a tool temperature reduction and a positive impact on the chip breakability process for HPC machining. Others have discussed and analysed the issue of the mechanism of tool wear during the high-speed machining of titanium alloys [21]. They showed that the tool life decreased with increasing cutting speed and improved productivity was achieved during high-pressure cooling. The high pressure of the cutting fluid causes the fluid to enter the slip zone, reducing the friction and temperature. A high-pressure coolant jet causes chip lifting, resulting in a reduced tool contact time and chip curl radius [19].

Attention is also paid to the aspect of the direction of the fluid supply to the cutting zone. Liang et al. [22] investigated the surface integrity of Ti6Al4V at different cooling pressures and injection positions. The researchers examined three injection positions, i.e., only the rake face injection, only the flank face injection, and both rake/flank face injection. They observed that, compared dry with HPC cutting conditions, the 3D surface roughness parameters decreased under high-pressure jet-assisted machining. Furthermore, Masek et al. [23] analysed the influence of the direction of the liquid supply to the cutting zone when machining with PCD tools. The investigation showed that when machining titanium alloys, dual cooling, at both the rake and the flank face, is highly recommended, and also the results illustrated that the appropriate HPC intensity was around 60 bar. The tool wear was reduced and the cutting edge was preserved. This resulted in an increase in the efficiency of the chip breaking process. In the literature, there are not enough research results on the effect of different values of the liquid delivery angle on the rake face of the insert in HPC turning.

Several described studies have focused on the optimisation and simulation of titanium alloy machining. For example, Grzesik et al. [24] presented an analysis of the tribological issues of the machining of the Ti6Al4V alloy, taking into account tool wear. Çolak [25] carried out the optimisation of HPC treatment using genetic algorithms due to the desired surface roughness. In addition, the finite element method for the titanium alloy machining simulation and optimisation process was described, taking into account the cutting force (*F_c_*), tool life *(T),* surface roughness *(Ra)*, material removal rate, and breakage of the chips [9,26,27,28]. Surface roughness and chip breakage were selected as optimisation criteria due to their importance to the finishing turning process. Physical difficulties in the machining of titanium alloys also translate into the complexity of computer simulation. The most frequently undertaken work in this area is related to the description of the calculation method or the adopted material model [29]. In turn, Moaz et al. [9] discussed the issues of the simulation of titanium alloy machining using various computer programs.

Due to the problems described above in obtaining the effective machining of titanium alloys, turning tests were carried out under HPC conditions. It should be noted that the presented research is a continuation of work [30] on the machinability of the Grade 5 ELI titanium alloy. The main aim of the previous analysis was to determine the areas of correct work of the chip breaker. In the current research, the direction of the cutting fluid was taken into account in the experiments. The aim was to increase our knowledge about the possibilities of improving the productivity and the chip breaking process when turning titanium alloys. The novelty was the analysis of the influence of the angle of liquid supply on the rake face of the cutting insert. The research plan was designed according to the Taguchi method and consisted of 18 test systems for different feed values (*f*) and directions (two angles α and β) of injection of the liquid stream on the rake face of the cutting tool. During the cutting force experiments, the cutting forces (*F_c_*, *F_p_*, *F_f_*) were recorded and microscopic analysis of the chips form was carried out at a constant pressure value *p* = 150 bar.

## 2. Materials and Methods

The mechanical properties and chemical composition of the workpiece (based on the technical data of the material supplier) are shown in Table 1. The longitudinal turning process under the conditions of cooling with increased pressure was analysed. Cutting tests were carried out for different directions of the coolant jet fed to the rake face of the cutting tool. The tests were conducted in longitudinal turning on a conventional lathe, equipped with a high-pressure plunger pump of 150 MPa pressure. A single nozzle of a specially adapted external machining feeding system was used. The angular position of the nozzle and the distance from the rake face were manually set with accuracy of ±2 degrees. A separate nozzle connected to the high-pressure hose was prepared. Clamps were used to ensure the position of the nozzle in the holder.

In the cutting tests, grade 1115 CNGG120404 cutting inserts with the SGF chip breaker and the Sandvik Coromant PCLNR2020K12HP toolholder were used. The radius of the corner of the cutting insert *r_ε_* = 0.4 mm was selected. The material was machined with a roller with a diameter *of Dc* = 50 mm. A constant cutting fluid pressure of *p* = 150 bar was used, and Blaser’s 10% Blasocut 2000 universal emulsion was used as the cutting fluid. A constant value of the cutting speed *v_c_* = 70 m/min and the cutting depth *a_p_* = 0.5 mm was assumed. The selected cutting parameters were within the range of those of finishing titanium alloys. The experimental research plan was developed according to the Taguchi method [31,32] for three variables, namely the feed *f* and two incidence angle values (α and β) of feeding for a jet of liquid onto the rake face of the cutting tool. The 18 test systems were designated. Each cutting test was repeated three times. Table 2 shows the accepted ranges of the cutting data values. The values of the cutting parameters were within the ranges of the cutting parameters recommended by the tool manufacturer.

The S/N ratio analysis strategy was adopted as “the lowest-best” according to Formula (1) [31,32].

(1) $$S/N=-10\cdot \log(\frac{1}{n}\sum_{i=1}^{n}y_{i}^{2})$$

Table 3 presents the test plan with the real values of the parameters used in the individual cutting tests.

**Figure 1:**
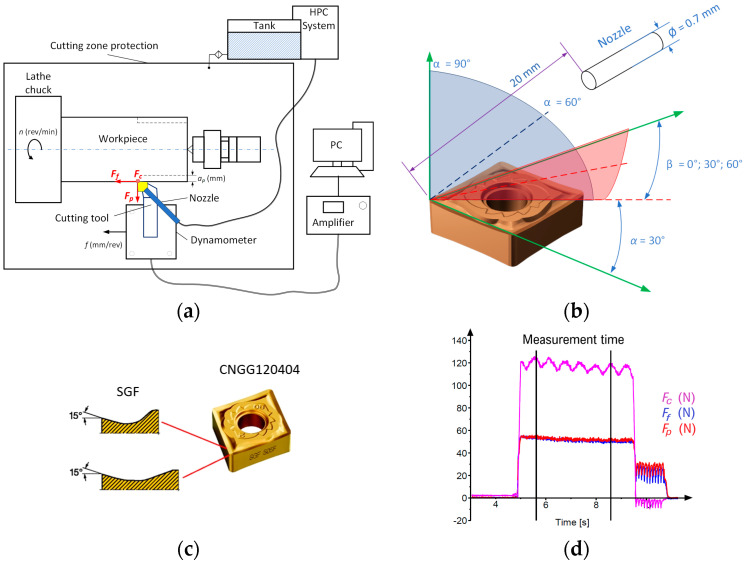
Measuring track system (**a**) general diagram, (**b**) setting of the nozzle feeding the cutting fluid, (**c**) dimensions of the SGF chip breaker, (**d**) example of the components of the total cutting force: *f* = 0.13 mm/rev, α = 60°, β = 60°.

During the research, measurements of the components of the total cutting force and microscopic measurements of the chip dimensions were carried out. To record and analyse the components of the cutting forces, a measuring track based on the 9257B dynamometer and the 5070B Kistler amplifier was used. A diagram of the measuring track together with the shape of the SGF chip breaker and an example of the recorded cutting forces are presented in Figure 1. Microscopic chip analysis was carried out using Keyence’s VHX-7000 3D, Keyence Corporation, Osaka, Japan microscope with dedicated measurement software.

It should be noted that the characteristics and classification of chips are inherently unclear or fuzzy [17]. In general, factors that affect the formation and shape of the chips are the cutting parameters, the shape of the rake surface of the cutting tool, and the way that the cutting fluid is delivered to the cutting zone. For this reason, an attempt was made to determine chips using geometric characteristics in accordance with their practical acceptability in industry [33,34]. In practice, a typical approach is to describe chips using terms such as excellent, good, weak, etc.

**Figure 2:**
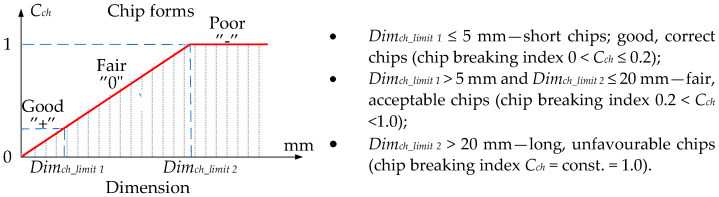
Chip classification method used in research.

The paper assumes modified characteristics of chips as presented by the authors of [33,35]. In general, chips can be described both linguistically and numerically according to rule (2), i.e.,

(2) $$Chip\_form=S_{N}\left({df}_{1},{df}_{2}\right)$$

where *N* = 1……4 represents four different types of chip shapes, i.e., arc/mass, spiral/circular, spiral/tubular, and ribbon, and *df_1_*, *df_2_* represent the two main dimensional characteristics of the chip, respectively. On the other hand, dimensional features converted into numerical values can be used to classify chips and determine their breakage coefficients [35,36]. In the research, the chip breakage index *C_ch_* (Figure 2) takes values from 0 to 1 and is described by the relationship (3). Lower *C_ch_* values represent better chip breakability.

(3) $$C_{ch}\left(Dimension\right)=\left\{\begin{array}{c} {Dim}_{ch}\;if\;{0<Dim}_{ch}<{Dim}_{ch\_limit\;2}\;\\ 1\;if\;{Dim}_{ch}\geq {Dim}_{ch\_limit\;2} \end{array}\right.$$

During the research, the analysis of the chip forms and their classification and evaluation were carried out. Over the research, only two forms of chips obtained during the machining tests were observed, i.e., arc- and spiral-type chips. For these types of chips, the dimensional characteristics were adopted according to Table 4. Based on the measured dimensions of the chips obtained, the chip breakage coefficient was determined according to Formulas (4)–(7).

(4) $${Dim}_{ch}=\bar{W_{ch}}+\bar{H_{ch}}=\frac{1}{n}\left(\sum_{i=1}^{n}{W_{ch}}_{i}+\sum_{i=1}^{n}{H_{ch}}_{i}\right)$$

(5) $${Dim}_{ch}=\bar{L_{ch}}+\bar{D_{ch}}=\frac{1}{n}\left(\sum_{i=1}^{n}{L_{ch}}_{i}+\sum_{i=1}^{n}{D_{ch}}_{i}\right)$$

where *n* is the number of chips.

(6) $$C_{ch}\left(W_{ch},H_{ch}\right)=\left\{\begin{array}{c} 0.01\cdot \left(\bar{W_{ch}}+\bar{H_{ch}}\right)\;if\;\bar{W_{ch}}+\bar{H_{ch}}\leq 1\\ \;1\;\left(constans\right)\;if\;\bar{W_{ch}}+\bar{H_{ch}}>1 \end{array}\right.$$

(7) $$C_{ch}\left(L_{ch},D_{ch}\right)=\left\{\begin{array}{c} 0.01\cdot \left(\bar{L_{ch}}+\bar{D_{ch}}\right)\;if\;\bar{L_{ch}}+\bar{D_{ch}}\leq 1\\ \;1\;\left(constans\right)\;if\;\bar{L_{ch}}+\bar{D_{ch}}>1 \end{array}\right.$$

**Figure 3:**
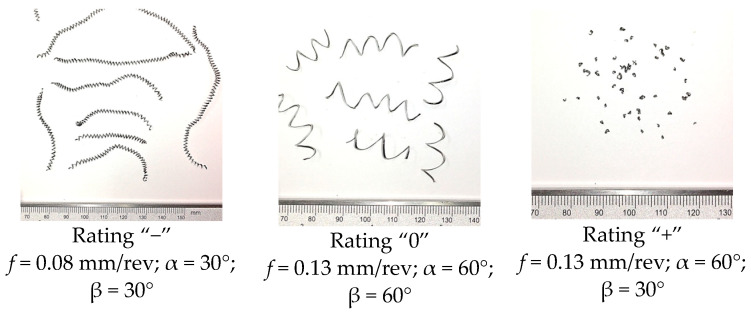
Sample photographs of chips obtained in cutting tests; constant parameters *v_c_* = 70 m/min, *a_p_* = 0.5 mm, *p* = 150 bar.

**Figure 4:**
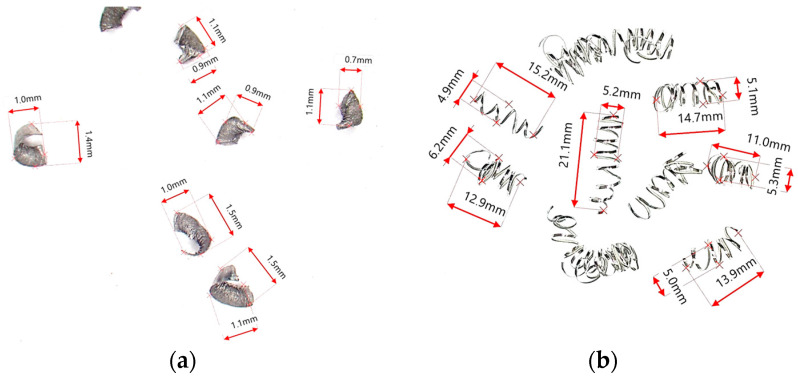
Examples of chip dimensions (**a**) for parameters *f* = 0.13 mm/rev; *a_p_* = 0.5 mm; α = 60°; β = 30°; (**b**) for parameters *f* = 0.08 mm/rev; *a_p_* = 0.5 mm; α = 60°; β = 0°.

In the cutting tests carried out, only long spiral or short arc chips were obtained. The main criterion for the assessment of chip form was the chip dimensions, i.e., length and height for arc chips or length and spiral diameter for helical chips. A three-stage assessment of the chip form was assumed, i.e., correct chips up to 5 mm long, acceptable chips up to 5–20 mm long, and unacceptable chips over 20 mm long. The following markings were adopted when assessing the form of the chips: “+”—chips correct (good); “−”—chips incorrect (poor); “0”—chips acceptable (fair). Examples of photographs of chips obtained during the cutting tests are shown in Figure 3. Figure 4 presents examples of the measurements of the dimensional features obtained in the chip tests.

## 3. Results and Discussion

According to the adopted research plan, the components of the total cutting force and the dimensions of the chips obtained were measured. The influence of the assumed variables, i.e., feed values *f* (mm/rev) and angles of the nozzle feeding the cutting fluid α (deg.), β (deg.) on the values of the components of the total cutting force, i.e., cutting *F_c_* (N)*,* feed *F_f_* (N) and passive *F_p_* (N), was analysed. Table 5 presents the results of the average values of the peripheral component of *F_c_mean_* and the breakage coefficient of *C_ch_mean_* chips, as well as the values of the *SN* parameter obtained in individual test systems. Table 6 and Table 7 present a statistical analysis of the test results.

Analysis of the measurement results showed a dominant linear dependence of the values of all components of the total cutting force on the feed value *f* when HPC was used to turn the Grade 5 ELI titanium alloy. Increasing the feed *f* = 0.08 mm/rev by 0.05 mm/rev caused an increase in the mean value of the *F_c_* component by approximately 77%. However, the angle of the nozzle applied to the feed liquid to the cutting insert rake face did not have a significant impact on the values of the components of the total cutting force.

**Figure 5:**
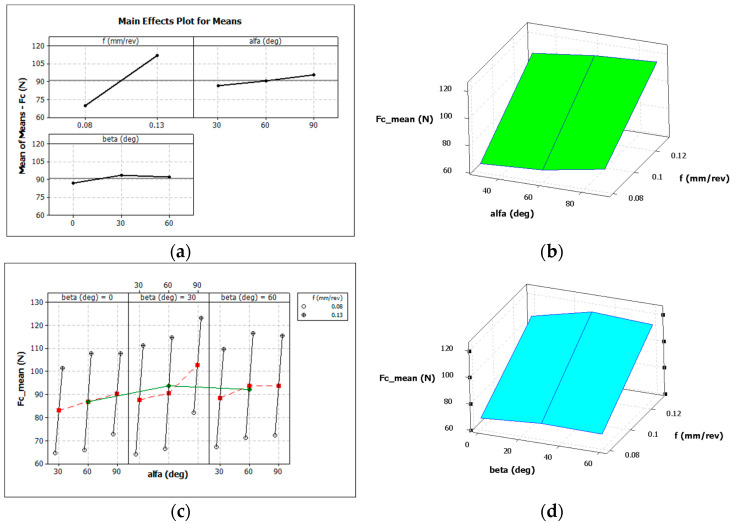
Influence of the analysed factors on the average values of the cutting force *F_c_*: (**a**,**c**) feed *f*, α and β angles, (**b**) only α angle and feed *f*, (**d**) only β angle and feed *f*.

Figure 5 graphically shows the influence of individual variables on the average value of the main cutting force *F_c_* for the case of the longitudinal turning of titanium alloy Grade 5 ELI.

**Figure 6:**
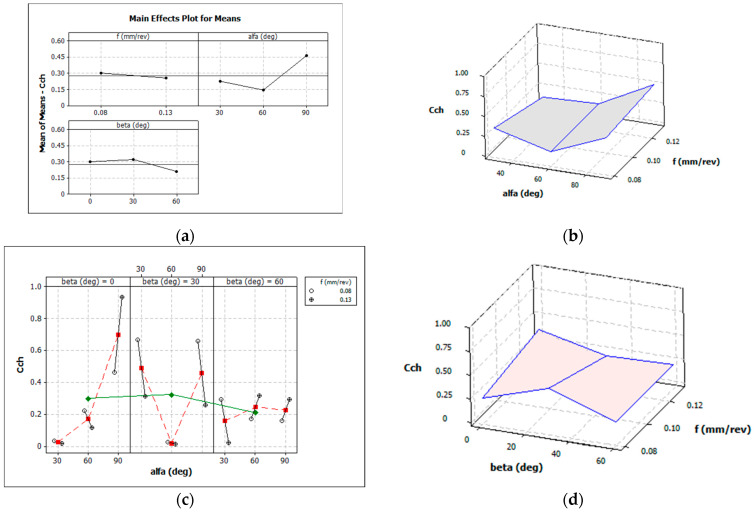
Influence of the analysed factors on the average values of the chip breakability index *C_ch_mean_*: (**a**,**c**) feed *f*, α and β angles, (**b**) only α angle and feed *f*, (**d**) only β angle and feed *f*.

Figure 6 graphically shows the influence of individual variables on the average value of the chip breakage coefficient *C_ch_* for the case of the longitudinal turning of titanium alloy Grade 5 ELI.

The analysis of the results of the data presented in Figure 6 and Table 7 does not indicate a clear influence of the angle of incidence of the cutting fluid on the chip breaking coefficient Cch. This can be explained by the fact that the use of the Taguchi method to find the best solution can be difficult. However, on the other hand, the measurements of the dimensions of the chips obtained and their form were varied. Therefore, to correctly interpret the results obtained, additional analyses were necessary, such as determining the chip breaker work area. Table 8 presents the evaluation of the form of the chips obtained in the experimental tests for the finishing range of the turning of Grade 5 ELI alloy.

**Figure 7:**
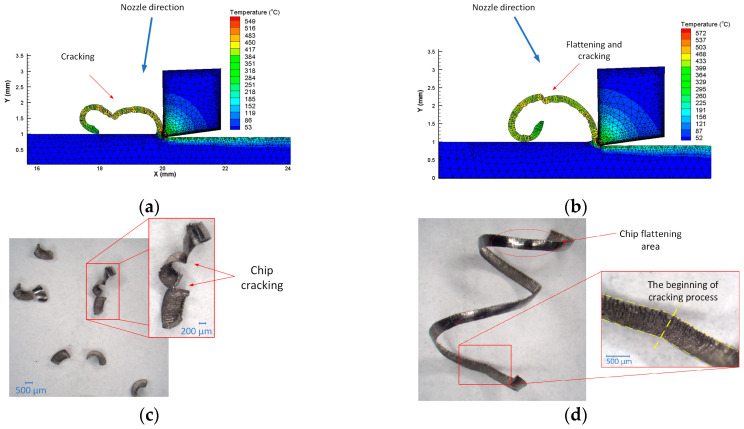
Examples of chip shapes obtained from process simulation and under real conditions: (**a**,**b**) process simulation for different cutting fluid directions, for feed *f* = 0.08 mm/rev, *a_p_* = 0.5 mm; (**c**) chip photographs for *f* = 0.13 mm/rev; *a_p_* = 0.5 mm; α = 30°; β = 60°; *p* = 150 bar; (**d**) chip photographs for *f* = 0.13 mm/rev; *a_p_* = 0.5 mm; α = 60°; β = 30°; *p* = 150 bar.

The analysis of the data (Table 8) obtained showed that the form of the chips in the longitudinal turning process was significantly influenced by the values of the angle of incidence of the liquid stream fed into the cutting zone. For feed *f* = 0.08 mm/rev, a favourable and acceptable chip form was obtained at an angle of α = 60° and angle values of β = 30° and β = 60°. Furthermore, a correct or acceptable chip form was obtained for angles α = 30° and β = 0° and α = 90° and β = 60°. On the other hand, for a higher feed value of *f* = 0.13 mm/rev, and thus a higher cross-sectional value of the cutting layer, more cases of obtaining a corrected chip form (more fine arc chips) were observed. The unacceptable form of the chips was obtained more often for increasing values of the α and β angles (generally for α = 90° and β = 60°). It could be seen that the form of the chips depended on the feed value. This was probably related to the increase in the strength of chips with a larger cross-section. Furthermore, for a higher feed value, the change in chip form could be influenced by an increase in the filling rate of the chip groove (chip reel) on the rake surface and an increasing contribution of the lift angle and height of the back wall of the chip breaker in the chip breaking process. The profile of the chip breaker used in the tests is shown in Figure 1c. For small feed rates, the chip groove may not be adequately filled with the workpiece material. This can cause a change in the mechanism of cracking and chip breaking. This process can also depend on the pressure of the liquid or the direction of its entry into the cutting zone. The cutting fluid fed to the outer surface of the chip causes the cracking of the chip by increasing the twisting of the chip, as well as flattening in the initial period of chip formation. Chip cracking occurs at the weakest points of the chip segments to be formed. Figure 7 shows an example the chip cracking process for different cutting fluid angles obtained from a computer simulation and photographs of chips with marked cracks and chip flattening area. To determine the influence of the angle of incidence of the cutting fluid on the cutting zone and the chip formation method, the turning process was simulated. The Johnson–Cook Equation (8) was used as the constitutive model. Table 9 presents the main model parameters and mechanical and thermal coefficients for the workpiece material.

(8) $$\sigma \left(\alpha ,\;\dot{\alpha ,}T\right)=(A+B\alpha^{n})(1+Cln\left(\frac{\dot{\alpha }}{\dot{\alpha_{0}}}\right))(1-{\left(\frac{T-T_{room}}{T_{melt}-T_{room}}\right)}^{m}$$

where *A* is the yield stress, *B* is the strain hardening coefficient, *C* is the strain rate dependence coefficient, *n* is the strain hardening exponent, and *m* is the material parameters of the temperature dependence coefficient. *T_melt_* is the melting temperature for the material, $\dot{\alpha }$ is an equivalent plastic strain rate, and $\dot{\alpha_{0}}$ is the reference strain rate.

HPC was modelled in the FEM software as a boundary condition in the form of the coolant pressure acting with different directions on the chip. In the simulation, a constant cutting fluid pressure was set: *p* = 150 bar. This is shown schematically in Figure 7a,b. The simulation showed the significant influence of the angle of incidence of the cutting fluid on the cracking mechanism of the chip and that the most vulnerable area of the formed chip was its segmentation sites. It should be noted, however, that the cutting process simulation was a 2D simulation and did not take into account the variable shape of the chip breaker profile. However, analysis of the simulation results showed that the chip breaking process occurred most often as a result of chips hitting the untreated surface. Similar conclusions were presented in [30]. The pressure acting on the outside of the formed chip and the direction of the treated fluid affect the change in the direction of the chip flow. This supports the chip cracking process. Chip breakage can occur in the area of chip flattening caused by the cutting fluid jet. Figure 7c,d show microscopic photographs of chips obtained from the research with marked fracture locations along chip segmentation and areas of chip flattening due to the working fluid pressure.

## 4. Conclusions

The purpose of this experimental research was to analyse the machinability of the Grade 5 ELI titanium alloy with tools made of cemented carbides under the conditions of finishing machining with the increased pressure of the cutting fluid. The main area of analysis was to determine the influence of the direction of the cutting fluid on the values of the cutting forces and the form of the chips obtained. The results of the analysis showed the following.

- The values of the components of the total cutting force depended linearly on the feed and did not significantly depend on the direction of feeding of the cutting fluid. The lowest cutting force values *Fc* were obtained for the feed *f* = 0.08 mm/rev. The angular direction of the cutting fluid feed caused a slight change in the cutting force *F_c_* value, i.e., on average, of several percent.
- The form of chips obtained (correct, acceptable, and unacceptable) depend on the range of feed values used and the angle of feeding of the cutting fluid to the cutting zone. The results indicate that the liquid feed angle does not have a significant effect on the value of the chip breaking coefficient and the low usefulness of the Taguchi method in finding the optimal nozzle setting. In practice, it seems to be a better solution to determine the working area of the chip breaker. Due to the form of the chips under the conditions assumed in the test, the optimal angular settings of the nozzle that fed the cutting liquid were α = 60° and β = 30°.
- Under HPC machining conditions, the process of forming and breaking chips depends mainly on the feed value. The chip breaker mechanism can also be influenced by the shape and degree of filling of the chip breaker on the rake face. The direction and method of feeding of the cutting fluid support both the winding and the breaking of the chips. Cutting fluid fed with an elevated strait on the outside of the formed chip can initiate the cracking and breaking process of the formed chip. There is synergistic action among the factors affecting the obtained form of chips, i.e., the assumed values of the cutting parameters (in particular, the feed), the degree of filling of the shape of the chip groove, and the direction and pressure of the cutting fluid. The direction of the liquid can support the cracking process of the chip. The set liquid pressure supports the chip breaking process by changing the direction of the chip flow. This results in the correct chip breaking cycle. Determining the correct angular position of the cutting fluid nozzle can be important for designers and tool holder manufacturers. Users of the currently used solutions can only slightly adjust (position) the nozzle dosing the cutting liquid (e.g., Iscar holders).

## Figures and Tables

**Table 1:** Mechanical properties and chemical composition—Grade 5 ELI.

Mechanical Properties		Chemical Composition (%)	
Tensile Strength	902 MPa	Al	6.10
Yield Strength 0.2%	815 MPa	V	4.13
Elongation	13%	Fe	0.05
Reduct. in Area	49%	C	<0.01
Hardness	29 HRC	N	0.01
		O	0.10
		H	0.003
		Ti	Remainder

**Table 2:** The variables’ values in the research plan.

Number	Coded Parameter	Real Parameter	Min	Value	Max
1	A	***f*** (mm/rev)	0.08		0.13
2	B	***α*** (deg.)	30	60	90
3	C	***β*** (deg.)	0	30	60

**Table 3:** Test plan with real values.

Number	A	B	C	*f* (mm/rev)	*α* (deg.)	*β* (deg.)
1	1	1	1	0.08	30	0
2	1	1	2	0.08	30	30
3	1	1	3	0.08	30	60
4	1	2	1	0.08	60	0
5	1	2	2	0.08	60	30
6	1	2	3	0.08	60	60
7	1	3	1	0.08	90	0
8	1	3	2	0.08	90	30
9	1	3	3	0.13	90	60
10	2	1	1	0.13	30	0
11	2	1	2	0.13	30	30
12	2	1	3	0.13	30	60
13	2	2	1	0.13	60	0
14	2	2	2	0.13	60	30
15	2	2	3	0.13	60	60
16	2	3	1	0.13	90	0
17	2	3	2	0.13	90	30
18	2	3	3	0.13	90	60

**Table 4:** Dimensional characteristics of chips obtained during cutting tests.

Group	Chip Shape	Dimension	Chip Index Characterisation
Arc/Bulky	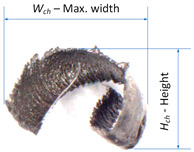	*W_ch_* (mm)	*C_ch_* (*W_ch_*,*H_ch_*)
		*H_ch_* (mm)	
Helical/Tubular	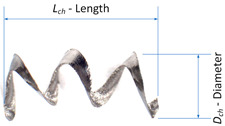	*L_ch_* (mm)	*C_ch_* (*L_ch_*,*D_ch_*)
		*D_ch_* (mm)	

**Table 5:** Results for measurements of cutting force *F_c_* and *C_ch_* (mean values).

Number	A	B	C	*f* (mm/rev)	*α* (deg.)	*β* (deg.)	*F_c_mean_* (N)	*F_c_min_* (N)	*F_c_max_* (N)	*S/N__Fc_*	*C_ch_mean_*	*C_ch_min_*	*C_ch_max_*	S/N*__Cch_*
1	1	1	1	0.08	30	0	64.7	55	70	−36.3	0.03	0.03	0.04	29.3
2	1	1	2	0.08	30	30	64.3	58	72	−36.2	0.67	0.55	0.82	3.4
3	1	1	3	0.08	30	60	67.3	61	74	−36.6	0.29	0.25	0.34	10.5
4	1	2	1	0.08	60	0	66.0	59	74	−36.4	0.23	0.19	0.27	12.9
5	1	2	2	0.08	60	30	66.7	60	73	−36.5	0.03	0.02	0.03	31.9
6	1	2	3	0.08	60	60	71.3	64	79	−37.1	0.17	0.15	0.19	15.3
7	1	3	1	0.08	90	0	73.0	68	80	−37.3	0.46	0.42	0.51	6.7
8	1	3	2	0.08	90	30	82.3	74	90	−38.3	0.66	0.58	0.76	3.5
9	1	3	3	0.13	90	60	72.3	65	79	−37.2	0.16	0.11	0.22	15.5
10	2	1	1	0.13	30	0	101.7	83	121	−40.2	0.02	0.02	0.02	35.0
11	2	1	2	0.13	30	30	111.3	103	119	−40.9	0.31	0.26	0.37	10.0
12	2	1	3	0.13	30	60	109.7	100	119	−40.8	0.02	0.02	0.03	32.6
13	2	2	1	0.13	60	0	108.0	100	118	−40.7	0.12	0.09	0.14	18.6
14	2	2	2	0.13	60	30	114.7	103	127	−41.2	0.01	0.01	0.01	37.6
15	2	2	3	0.13	60	60	116.7	108	125	−41.4	0.32	0.29	0.36	9.9
16	2	3	1	0.13	90	0	108.0	101	114	−40.7	0.94	0.84	1.03	0.5
17	2	3	2	0.13	90	30	123.3	113	133	−41.8	0.26	0.22	0.30	11.7
18	2	3	3	0.13	90	60	115.7	108	123	−41.3	0.29	0.23	0.36	10.5

**Table 6:** Analysis of variance for mean values—main force *F_c_*.

Source	*DF*	*Seq SS*	*Adj SS*	*Adj MS*	*F*	*P*
*f* (mm/rev)	1	8064.5	8064.5	8064.5	8064.5	0.000
		8064.5	8064.5	8064.5	8064.5	
		8064.50	8064.50	8064.50	8064.50	
		595.19	595.19	595.19	595.19	
*α* (deg.)	2	259.6	259.6	129.68	9.58	0.003
		259.6	259.6	259.6	259.6	259.6
		129.80	129.80	129.80	129.80	129.80
		9.58	9.58	9.58	9.58	9.58
		0.003	0.003	0.003	0.003	0.003
*β* (deg.)	3	155.8	155.8	77.91	5.75	0.018
		155.8	155.8	155.8	155.8	155.8
		77.91	77.91	77.91	77.91	77.91
		5.75	5.75	5.75	5.75	5.75
		0.018	0.018	0.018	0.018	0.018
Residual Error	12	162.6	162.6	13.55		
Total	17	8642.5				
Regression Equation	*F_c_*(*f*,α,β) = −9.87 + 847·*f* + 0.155·α + 0.088·β; *R-Sq* = 97.3%, *R-Sq*(adj) = 96.7%					

**Table 7:** Analysis of variance for mean values—chip breakability index *C_ch_*.

Source	*DF*	*Seq SS*	*Adj SS*	*Adj MS*	*F*	*P*
*Regression*	8	0.6309	0.6309	0.0789	1.36	0.326
*Linear*	3	0.2015	0.2015	0.0672	1.16	0.377
*f*	3	0.0095	0.0095	0.0095	0.16	0.695
*α*	1	0.1687	0.1687	0.1687	2.91	0.122
*β*	1	0.0232	0.0232	0.0232	0.40	0.542
*Square*	2	0.1769	0.1769	0.0885	1.53	0.268
*α*	1	0.1582	0.1582	0.1582	2.73	0.133
*β*	1	0.0187	0.0187	0.0187	0.32	0.584
*Interaction*	3	0.2525	0.2525	0.0842	1.45	0.291
*f α*	1	0.0603	0.0603	0.0603	1.04	0.334
*f β*	1	0.0095	0.0095	0.0095	0.16	0.695
*α β*	1	0.1827	0.1827	0.1827	3.16	0.109
Residual Error	9	0.5211	0.5211	0.0579		
Total	17	1.1519				
Regression Equation	*C_ch_*(*f*,α,β) = 0.9957 − 5461·*f* − 0.02745·α + 0.171·β + 0.0002·α^2^ − 7.6·10^−5^·β^2^ + 0.0945·*f·*α − 0.0375·*f·*β − 1.68·10^−4^·α·β *R-Sq* = 54.8%, *R-Sq*(adj) = 14.6%					

**Table 8:** SGF chip breaker work area and chip form assessment.

	*f* = 0.08 mm/rev			*f* = 0.13 mm/rev		
	*β* = 0°	*β* = 30°	*β* = 60°	*β* = 0°	*β* = 30°	*β* = 60°
*α* = 30°	+	−	−	+	−	+
*α* = 60°	−	+	0	0	+	−
*α* = 90°	−	−	0	−	−	−

**Table 9:** Simulation model parameters and mechanical and thermal coefficients for titanium alloy.

Material Constants		Mechanical and Thermal Coefficients	
*A*	997.9 MPa	Thermal Conductivity	6.6 W/m·°C
*B*	653.1 MPa	Heat Capacity	526 J/Kg·°C
*n*	0.45	Density	4430 Kg/m^3^
*C*	0.0198	Young’s Modulus	1.1 × 10^11^ Pa
*m*	0.7	Poisson’s Ratio	0.31
*T_melt_*	1277 °C		

## Data Availability

Data available in a publicly accessible repository.
